# Revisiting our own professional identity in order to move midwifery forward in coherence

**DOI:** 10.18332/ejm/201991

**Published:** 2025-05-05

**Authors:** Raymonde Gagnon, Céline Lemay

**Affiliations:** 1 Midwifery Department, University of Québec TroisRivières, Trois-Rivières, Canada

**Keywords:** midwifery, community-based participatory research, professional identity, Quebec

## Abstract

**INTRODUCTION:**

The midwife community of Québec, expressed the need to revisit the identity markers of the profession after been integrated for over twenty years into a medical and hospital-centered healthcare system. There was a need to identify meaningful concepts and principles that allow them to practice in coherence with their uniqueness and in continuity with their historical roots.

**METHODS:**

Qualitative research with a cooperative inquiry approach was chosen. A total of 65 midwives from various practice settings met during two days of reflection. Focus groups of parents (15) where added in order to enrich midwives’ discussions.

**RESULTS:**

Five themes emerged: the concept of becoming a mother as a process of transformation, the accompaniment of this process, the concept of giving birth, the professional posture, and distinctive aspects from the medical paradigm. Parents emphasized the importance of a family-centered approach, trusting relationship, choices, and continuity.

**CONCLUSIONS:**

A reflective and collective process has allowed to reconnect with the meaning of being a midwife and practicing in coherence with its specificity. Some questions remained in relation to feminism, the spiritual dimension of birth and the community engagement. Moreover, the abundant use of the term ‘physiology’ for childbirth does not correspond to the richness of practice narratives and obscure the ineffable sacred character of birth. Midwives face the challenge of integration and inter-professionalism without losing parts of their autonomy and their professional voice. It would be appropriate to revisit the basic training program and examine the elements put in place to build and reinforce midwifery professional specific identity.

## INTRODUCTION

The resurgence of midwifery practice in Québec, Canada, took root in the 1970s and 1980s, and is linked to social movements where women wanted to reappropriate the events surrounding birth^1^. A group of midwives formed and developed a strong identity based on shared values, the importance of women, a concept of pregnancy and childbirth, and an anchoring in the community world^2^.

The profession was recognized legally in 1999 as an autonomous profession in the healthcare system of Québec. Midwives are giving maternity care with their own responsibility and the women they follow also have the choice of birth place: οn 80–85% in a birth center, 2–3% in hospital, and 15–20% at home, out of 3659 births in 2024.

This transition from a community practice (1970s and 1980s) to a professional practice created an identity crisis that mobilized them to reflect on and define the hallmarks of their specificity^2,3^.

Over twenty years after its legalization, the profession is expanding and most practicing midwives have inherited the philosophy of the profession, the bearer of meaning par excellence, without having participated in its elaboration.

Midwives face the challenge of integrating into a healthcare system structured by medical perspective on maternity and childbirth, fragmentation of care, management, performance and standardization. Since their practice is fundamentally about normalcy and health, continuity, woman-centered care, shared decision-making, and choice of birthplace, it creates tension and disturbs their sense of their professional identity. A consequence like abdication of professional voice^4^ can erode the quality of midwifery model of care promoted by WHO in 2024^5^.

As they felt that their professional identity was misunderstood by other healthcare providers and even by the public, members of the professional association of midwives, the *Regroupement les Sages-Femmes du Québec* (RSFQ), asked to set up a committee to examine the elements of the profession’s specificity, highlighting its originality and relevance^6^. At the same time, a consumer’s voice from the community expressed the need to reaffirm the importance of having access to midwifery services according to a model that corresponds to the expectations of women and couples^7,8^.

This problem led to two questions: ‘How can we understand the identity hallmarks of Québec midwives roots over 20 years after the profession’s integration into the public healthcare system?’ and ‘How can we hear the voice of the midwives’ clientele on what they consider as “unique” in this profession?’.

The aim of this research was to identify meaningful concepts that allow midwives to express their specificity and practice in continuity with their historical roots, in coherence with their fundamental principles, affirming their identity as a unique profession in Québec society in the healthcare system.

## METHODS

### Study design and research team

We conducted qualitative research using a participatory approach, embodying the collaborative spirit of Québec midwives. The ‘cooperative inquiry’ method was employed to engage midwives in reflecting on their professional identity and co-constructing its essential elements^9-12^. This method involves individuals with shared concerns developing new perspectives and meanings from their experiences^13^. It is a powerful way to democratize knowledge creation by transforming experience into formalized ‘propositional knowledge’^10^ through cycles of reflection, dialogue, and reflexivity^10^,^12^,^14^-^16^.

The research team comprised two midwife researchers, a philosopher and ethicist whose work focuses on issues of professional identity, a psychologist specializing in professional practices and a midwife representing the RSFQ, and none were in a hierarchical position relative to the participants. The project was evaluated by the Ethics Committee of the Université du Québec à Trois-Rivières (certificate number: CER-19-253-07.08) before its start. Each participant including parents signed an information and research consent form. We report the study using the Consolidated Criteria for Reporting Qualitative Research (COREQ)^17^.

### Participants and conduct of the research

Initially, the administrative staff of the RSFQ sent an email invitation to all midwives from its member list to participate in two days of reflection as part of this research. The inclusion criteria were: being in active practice or on temporary leave, and retired midwives. We also invited parents users in order to understand what they found unique about midwives. We ensured that all the regions of the province where represented.

On the first day, discussion in small groups took place on various topics and questions. Among these, they were asked to recount lived experiences, to talk about the ideal they were seeking in becoming a midwife, and whether this had become a reality, or to present midwife services to a woman who would like to know more and to react to scenarios, e.g. a woman who wants an epidural, who refuses screening tests or who does not want an induction. Pre-trained research assistants led the discussions, which were followed by a plenary session. After the first day, the participants were encouraged to do a mini online assignment where they had to propose the best way for them to represent a midwife, either by an image, a photo or a drawing accompanied by explanations about their choice.

We conducted two focus groups with parents using midwives’ services. It was important to include both parents and midwives’ perspectives, as identity is also shaped by those who receive the services. Interested parents replied to a user group network invitation by e-mail and received an explanatory form and consent authorization before participating in the focus group.

### Data analysis

Discussions were digitally recorded and transcribed. The data were first coded individually by three of the researchers and then discussed to enable triangulation. An initial thematic analysis was carried out with the first data collected with the midwives and parents.

Secondly, during the 2nd day of reflection with the midwives, we were able to share the results obtained, deepen the reflection and co-construct together around shared professional values.

An in-depth analysis of all these results enabled us to identify the main themes relating to the identity and specificity of midwives in Québec. The analysis process is illustrated by examples in Table 1.

**Table 1:** The concept of giving birth, the analytical process, Québec, 2021 (N=65)

*Criteria components*	*Meaning unit*	*Corresponding attitudes*	*Meaning unit*
**Ability to give birth to her child**	*‘Because she had the impression that this one came from her, because she had really brought it into the world herself, with all her ability, strength and power as a woman.’* (Participant 6)	**Recognizing a woman’s ability**	*‘To trust that women know that they are capable of achieving it because there is something inside them which is stronger and which arises spontaneously.’* (Participant 6)
		**Trusting in the power of women**	
**The sacredness of childbirth**	*‘The welcome … into the sacred, into their total intimacy.’* (Participant 39)	**Being a guardian of the sacredness of birth in ordinary life**	‘I find that the fact of being the guardian of this polarity between something magical, magnificent, extraordinary, but really banal as because babies are born every day and there is something I find super …’ (Participant 51)
	*‘What moves me most in our work is when we witness, participate in, the sacredness of physiology ... When women have the space to move.’* (Participant 35)		*‘I’d add an element that coexists, that’s fundamental for me, and that’s to be the guardian of the sacred ... That translates into a set of gestures, words and silences, respect, all that, in a dance because that’s the basis ... it’s important that I remember it every time, because in the whirlwind of going fast to a birth, I have to speak to myself and say: OK, settle down. You’re going to a place where the sacred happens ...’* (Participant 56)
**Physiological processes**	*‘I find we’re in there, in maximum physiology, and you have to trust when the woman is in there.’* (Participant 12)	**Being a guardian**	‘*Respect for physiology and the guardian of physiology ... You’re the keeper of the bubble.’* (Participant 1)
		**Protecting the space**	*‘I try to arrive as calmly as possible so as not to break the bubble.’* (Participant 12)
**Spirituality**	*‘The times when I really felt like a real midwife were when things were happening that were out of my control, but also out of the woman’s control. That it was happening on a higher level than that, but that there was also something protecting us and being there for us, to influence what was happening.’* (Participant 2)	**Recognizing the greater than us**	*‘And to witness that which is greater than ourselves. You know, you talk about the sacred, you talk about what we can’t explain, what we don’t understand ... we have the privilege of witnessing things that can’t be explained, and of having the openness in our hearts and the space to recognize them.’* (Participant 50)

## RESULTS

Sixty-five midwives, nearly a third of the eligible midwives, participated in the discussion workshops. The focus groups with the parents reached 15 participants (14 women and 1 man), ultimately representing the 12 birthing centers and service settings.

### Identity markers of Québec midwives

The identity markers of today’s midwives are mainly reflected into five themes: their concept of becoming a mother as a process of transformation, the accompaniment of this process, the concept of giving birth, their professional posture, and what makes them a profession distinct from the medical paradigm. In addition, the data allowed us to identify the need to express their professional identity in words that are meaningful to them and understandable to the public and decision-makers.

### The concept of becoming a mother

This component is the most fundamental of midwives’ identity. Midwifery practice goes beyond just helping women to give birth. Becoming a mother involves the continuum of pregnancy, childbirth, postnatal period, and breastfeeding. This phenomenon is ‘*matrescence*’. It is considered a unique process of transformation that belongs to women. It is guided by meaning and not only by facts. Midwives talk about being there to help welcome the baby and the new family from a perspective of empowerment, respect for the woman’s transformative experience and an awareness that it is also a social process. All verbatims have been anonymized using fictitious names. We give an example:

*‘To take them where they are, no judgement. Then to move forward with them, to progress, to journey. To see them being born I would say ... in any situation there, you know.’* (Judith)

Midwives talked about being ‘guardians of the possible’, that is, they let things emerge as they come:

*‘So give the space they need to live through maternity.’* (Isabel)

### The accompaniment of the processes

If the woman is considered in her ability to carry and give birth to her child, it makes sense for midwives to talk about the accompaniment of the processes. It is clear that we go further than a technical practice. Midwives talk about knowing how to ‘be there’, in listening to and respecting each one. The accompaniment includes confidence in women and mobilization of knowledge and skills in the service of welcoming life and ensuring safety:

*‘It’s really with the personality of each woman, but also in each situation. And to be able to accompany a woman by letting her define herself.’* (Mila)

### The concept of giving birth

Midwifery practice is based on the recognition of the ‘ability to give birth to one’s child’ and the power of the woman. It is about trusting this force. A participant indeed spoke of it in these terms:

*‘…because she had the impression that the baby came from her, because she had really given birth to it herself, with all her capacity, strength and power as a woman.’* (Alyson)

Midwives talk about childbirth as a ‘physiological process’, as a ‘powerful event’ and even its ‘sacred character’. They see themselves as ‘guardians’ of the process and ensure to ‘protect the space’ proper to its development. They emphasize respect for physiology by considering themselves a ‘guardian of physiology’ and also ‘guardian of the sacredness of birth in ordinary life’. Moreover, even if discrepancies have been observed regarding the recognition of the spiritual dimension of birth, midwives still agree to recognize ‘the greater than us’:

*‘…being a witness to what is greater than us. You know you talk about the sacred ... We have the privilege of witnessing things that can’t be explained, and having the openness in our heart and the space to recognize them.’* (Lucie)

### The midwife as a professional

The profession is essentially defined by the relationship to the other rather than by actions. This is intended to be egalitarian and is focused on the needs of each woman. It is impossible to generalize. It is based on the meeting of two different types of knowledges and it evolves in a mutual trust that is woven as the follow-up progresses. That is why the midwives have talked about the importance of continuity, of taking time and of commitment to the relationship:

*‘A relationship of trust requires a relationship. So if you see a different person every time, there is very little or no relationship.’* (Edith)

Moreover, the competence of midwives is understood not only as technical skills but much more that of knowing how to prioritize things in practice. It is a question of discerning what is essential from what is less so while maintaining a holistic approach. It is about knowing how to intervene effectively when necessary. The action is as much through words and language as through gestures.

### Distinctive aspects of the midwife’s identity in relation to the medical paradigm

Without placing them in opposition, three elements distinguish the midwife’s identity in relation to the medical approach. The first is confidence, the second is the recognition of the woman’s autonomy and the third is knowledge.

Confidence is a fundamental characteristic of the midwife’s professional posture, deeply rooted in a health perspective. Normality is an *a priori* state, which contrasts with the medical perspective which only considers normality in retrospect and which considers that the woman necessarily needs care and interventions in order to give birth. Risk is certainly part of the conversation but the goal is not about its elimination. The uncertainty of any situation is assumed:

*‘We don’t have the same glasses.’* (Jane)

Furthermore, the partnership with women involves the meeting of two different visions and perceptions of risk. On the one hand, the midwife can no longer adopt the stance of an expert who holds the truth and expects consent rather than facilitating genuine choice. On the other hand, an autonomous woman can make decisions and choices based on her needs and values. Her perspective, including her perceptions, experiences, and plans, must be taken into account. The choice of birthplace is a fundamental aspect of this autonomy.

This project can only be defined by the woman who will specify her trajectory at each of the decisions made during the process. In order to adapt in an optimal way to the woman from a perspective of empowerment, midwives consider that their professional autonomy is central to the profession because *‘that’s how we will serve women better’*.

Finally, the knowledge acquired through training is not there to be predominant and to impose on women an experience of dispossession or technical invasion. It differs from the medical paradigm as much by the knowledge coming from other disciplines as by other ways of knowing such as experience, intuition and shared stories:

*‘We are just two women and that we are in a relationship, and that yes, I may have knowledge but I want to share it*

*… What you tell me is as good as what I tell you.’* (Esther)

### The parents’ point of view

Parents have revealed what was most significant in their experiences of being taking care by a midwife. These elements are: the family approach, informed choices in a context of autonomy and empowerment, continuity of the relationship, caring for the person, a relationship of trust, availability and choice of place of birth.

The relationship that is established and developed with the midwife is described as a relationship between:

*‘two human beings who meet and journey through different stories.’* (Jasmine)

Time and availability help the creation of a meaningful and human relationship, which is considered important for building trust.

Parents wish that midwives maintain their orientation towards physiological childbirth and that they remain a strong profession by being close to women:

*‘They help mothers to be born, not just babies.’* (Emy)

Some parents express fear of the medicalization of the profession. Others recognize their significant presence in society, beyond what institutional language calls high quality and personalized services:

*‘They will still bring a lot of beauty into our world, as they have helped us to transform and welcome our baby.’* (Sara)

Interestingly it was the parents who have referred to feminism in relation to childbirth:

*‘I find it deeply feminist in fact to be able to give birth … in our power and in our nature, while we really feel infantilized (in the medical field).’* (Claudie)

Subsequently, we questioned the participating midwives on this subject, since they had not mentioned it on the first day. Even though the vast majority accepted feminism in relation to childbirth, many question the meaning of feminism and its implication for midwifery practice.

## DISCUSSION

Several studies have already explored the professional identity of midwives^18,19^, but the current research was essential to gain a deeper understanding of the unique aspects of the midwifery profession in Québec and to facilitate a collective process of appropriation.

Midwives have been integrated into changing society and healthcare system. Moreover, despite discourses affirming that pregnancy and childbirth are normal life processes^20^, the ideology of risk, the use of technology and medical interventionism are more dominant than before^21,22^. Midwives are in flux but this research was about identifying what is still solid in the hallmarks of their professional identity and what is different.

### The importance of accompaniment

Beyond birth, it is the concept of becoming a mother as a continuum encompassing the three periods of the maternity experience (before, during and after birth) that is most significant for midwives. Practicing within the framework of complete follow-ups, shapes this representation and makes sense with the lived experience of women which is continuous.

The recognition that it is a process of transformation places midwives in a role of accompaniment that requires relational skills and not only clinical ones to play their role with women and families. A relationship of mutual trust is at the heart of this practice.

Finally, the singular aspect of the maternity experience makes accompaniment essential to grasp the specific needs of each woman and each family, as highlighted by the parents during the focus groups, and also in various studies, in particular that of Makarova et al.^23^.

### Physiology in the language

The participating midwives recurrently used the term ‘physiology’. And this, as if it were the only dimension of childbirth whereas their language, when they talk about their professional world, reflects a much broader, richer and deeper concept of childbirth. This is all the more surprising since this term was absent from the language of midwives in the 1980s and 1990s^24^, and that twenty years earlier they rather used the term ‘natural childbirth’. It should be noted that the terms ‘physiological childbirth’, ‘natural’ and ‘normal’ have multiple concepts and definitions that overlap and confuse, depending on the organizations that define and promote them to the public, such as the Canadian Association of Midwives^25^, and Maternity Care Working Party^26^.

Recognizing, valuing and being a guardian of the physiological processes of the female body is important to be declared *a priori* by midwives. However, speaking only from the biological dimension of giving birth is a sign of poverty of language to account for this human experience, the foundation of our lives and of any society.

Why do not midwives use a language that corresponds to what they experience, understand and conceive of giving birth? What would be the perceived or felt barriers to them using a language that represents them? A midwifery language would be a means of affirmation and would allow emancipation from the weight of biomedical and institutional language.

### Intervention and non-intervention

Midwives’ relation to intervention in childbirth seems paradoxical. On the one hand, they acknowledge that medical pressures and the fear of legal procedures are factors leading them to do more interventions without necessarily using their professional judgement and autonomy to do the most appropriate action with each woman. On the other hand, they talk about the ‘invisible work’, of being a ‘guardian of the processes’, of ‘holding space’ and of ‘protecting’ the space of each unique unfolding process. They talk about patience, taking time and trusting. It is consistent with the position of the International Confederation of Midwives regarding the promotion of non-intervention in normal childbirth^27^ as well as having a ‘guardian’ role^28^.

It seems that it is through the relationship that midwives are able to prioritize the best actions in each situation and context. Midwives will eventually have every interest in clarifying how, as a relational profession, they conceive ‘midwife intervention’, a specificity that enables them to support women and families by modulating intervention and non-intervention. Their way to express it clearly will give opportunities for better dialogue with parents, other professionals in the healthcare network, and society in general.

Therefore, the professional autonomy of midwives deserves to be assumed, encouraged, consolidated so that they can completely fulfil their role for the benefit of women and families who call upon them.

### Relation to feminism

While 20 years before, midwifery practice was part of the feminist movement, this link and this posture are far from being as clear today. Although they still consider it important to promote the empowerment of women and their experiential knowledge, it was the women themselves, during focus groups, who saw childbirth as being a profoundly feminist act. When confronted with these remarks, some midwives have shown discomfort and seem to believe that such a qualification could affect negatively the image of the profession. Do they think it is outdated, too radical or leaves no room for men?

Yet midwives constantly refer to their philosophy which clearly express the principles of feminist intervention^29^. By being on the side of women, the profession will not be able to ignore this aspect of their professional identity. It seems that this fundamental element of their practice is neither conceived nor consciously linked to a feminist posture.

The teaching body, whose mission is to train competent midwives who know how to embody a practice consistent with the specificity of their profession, could strengthen the understanding of these links.

### Implications

The research methodology and participatory approach made it possible to revisit the identity markers of midwives twenty years after the legalization. It fostered openness to exchanges focused on the most significant aspects of the profession. Several midwives said they felt privileged to take time out to address these issues while strengthening their sense of belonging to the midwifery community. Even if points of view can sometimes diverge, there is always a hard core around the deep values that drives the profession and that are likely to nourish the daily life of midwives.

In addition, this approach led to exchanges enriched by the research results which have been retransmitted as desired by the RSFQ to all decision-making bodies of the profession. Several presentations, always with a subsequent discussion component, were made to the boards of directors and strategic committees of the RSFQ and the *Ordre des Sages-Femmes du Québec* as well as to the heads and professors of the midwifery training program at UQTR. Then, webinars were offered to all midwives and midwifery students to share the results and stimulate their reflections. The RSFQ, as its president told us, also uses some of the results in its representations to policy makers and other collaborative partners in their professional and health network. Recently, the RSFQ has also completed the drafting of a statement of principle on the specificity of the profession largely inspired by these results.

Québec Midwifery faces a number of challenges: clarifying the relation with feminism, with community engagement and finding words that resonate with the ineffable sacred character of birth. How to express that it is ‘greater than us’? How also to use a language that expresses the very meaning of what characterizes the professional action when it comes to making midwifery known to the public and to the health system authorities? Appropriating the specificity of the profession is a powerful tool for practicing midwifery in a way that is consistent with its principles. Midwives will be better equipped to face the challenge of integration and inter-professionalism without losing some of their autonomy or experiencing the abdication of their professional voice^30,31^. It would be advisable to revisit the basic training program and examine the elements put in place to reinforce professional identity and specificity.

### Strengths and limitations

The main strength of this research has been to allow the appropriation of results because of the experience of living its process. Similarly, the interpretation of the results by an interdisciplinary team has contributed to highlight them in the social and medical context in which midwives evolve. Such research cannot reach all midwives either out of interest or availability. Similarly, we would have liked to have even more parents in the discussion groups. These elements are limitations to the generalizability of the results. However, the fact that the people present came from various regions of Quebec and that at the time of the discussions, we mixed these participants made it possible to bring out the main points of view around the questions addressed.

## CONCLUSIONS

This participatory research of collective reflection has allowed midwives to name the fundamental elements of their professional identity and to specify their uniqueness in the landscape of maternity care in Québec. Several elements have been identified: the concept of childbirth continuum as a singular process of transformation; the role of accompaniment and guardianship of the possible. Midwives talk about ‘holding the space’ and the ability to ‘be there’. They consider not only that women are capable of giving birth to their child but that childbirth is a normal process *a priori*, clearly distinguishing themselves from the medical posture regarding childbirth. It also reveals a professional posture as a relational practice rather than a technical one, articulated by a health approach where trust, autonomy of decisions and empowerment are mutually reinforcing each other. The relation to risk and uncertainty is assumed, and the midwife draws on different areas of knowledge in her professional judgement. Parents appreciate what midwives bring them: the family approach, choices, continuity, and trust. At the same time, they are concerned about the medicalization of the profession.

## Data Availability

The data supporting this research are available from the authors on reasonable request.
